# The effect of cessation of drinking water fluoridation on dental restorations and crowns in children aged 3–5 years in Israel – a retrospective study

**DOI:** 10.1186/s13584-024-00637-5

**Published:** 2024-09-20

**Authors:** Shiran Shemesh Nezihovski, Mordechai Findler, Tali Chackartchi, Jonathan Mann, Doron Haim, Guy Tobias

**Affiliations:** 1 Department of Community Dentistry, Faculty of Dental Medicine, Hebrew University of Jerusalem, Hadassah Medical Center, Jerusalem, Israel; 2 Dental Research Unit – Maccabi-Dent, Maccabi Healthcare Fund, Tel-Aviv, Israel; 3 Department of Periodontology, Faculty of Dental Medicine, Hebrew University of Jerusalem, Hadassah Medical Center, Jerusalem, Israel

**Keywords:** Community water fluoridation, Public health, General anesthesia, Health policy

## Abstract

**Background:**

Community water fluoridation began in the 1945 as a public health measure to prevent and control caries and was implemented in Israel in 1981. Community water fluoridation reduced caries significantly, but in 2014, the Ministry of Health decided to stop Community water fluoridation in Israel. The aim of our study was to examine the effect of fluoridation cessation on the dental health of children aged 3–5, treated in “Assuta Tel Aviv” dental clinics, under general anesthesia or deep sedation.

**Methods:**

The computerized Maccabi-Dent database provided data for this retrospective study. Records from the years 2014–2019 including treatment codes for procedures relevant to the study, the number of stainless-steel crowns and restorations of all types were examined. Kruskal-Wallis test was performed to compare the results from before and after fluoridation cessation.

**Results:**

A statistically significant increase in the mean number of treatments in the years after fluoridation cessation (*P* < 0.05) was found. There was approximately a two-fold increase in the number of all treatments for all ages.

**Conclusion:**

The results of the study emphasize the advantages of water fluoridation and are further proof of the need to restore community water fluoridation in Israel.

## Background

### Oral diseases

Microbial diseases of the oral cavity affect the hard (caries) and soft tissues (periodontal diseases). Periodontitis is a progressive and chronic disease impacting the tooth supporting structures and may eventually lead to tooth loss [1].

Dental caries in permanent teeth is the most common health condition worldwide according to Global Burden of Disease 2019 [2]. Its earliest detectable stage is the appearance of a white spot on the tooth, which may later cavitate to the extent that the tooth is lost. It may cause pain, difficulty eating, a reduction in life quality, and in severe cases, hospitalization. This multifactorial disease, is due to an imbalance between dietary sugars, the dental biofilm, and the host, within the oral environment, causing de-mineralization of tooth enamel [3]. Some periods in life are more prone to this imbalance, such as the stages of primary and mixed dentition [4]. Prevention of the disease by using fluoride, removing dental biofilm and reducing sugar intake [3], is preferable but once a lesion is cavitated, the first stage of treatment is removal of the affected tooth material and placing a restoration. When too much tooth structure has been lost due to caries to retain a restoration a crown may be required [5], in pedodontics prefabricated stainless steel crowns are used [6].

### Fluoride and its effect on enamel

Fluoride is a naturally existing anion found in minerals, rocks, and soil. All water sources contain fluoride, and levels depend on the specific geographical conditions. Fluoride concentration in rivers, streams, or wells is usually below 0.5 mg/l [7].

There is a strong affinity between fluoride and biological apatite (the main mineral component of teeth). The hydroxyl component of calcium hydroxyapatite of tooth enamel is easily replaced by fluoride, becoming fluorapatite, which is less soluble by acids. Fluoride in dental plaque and saliva, inhibits demineralization and promotes remineralization of initial caries lesions [8], interferes with bacterial glycolysis, and at higher concentrations fluoride is bacteriocidic.

### Water fluoridation

Fluoride can be used to prevent caries in 3 ways: (1) community-based – provision of fluoridated water, salt, and milk, (2) professional administration of fluoride varnish and gels in the dental clinic, (3) self-administered – using toothpastes and mouthwashes [7].

Community water fluoridation has been used as a public health measure to prevent and control caries since 1945, following the epidemiological studies of H. Trendley Dean et al. (1942) [9], which found the optimal level of 1 mg/l of fluoride that provides maximum protection against caries, with minimal dental fluorosis.

Community water fluoridation involves adjusting the amount of fluoride in water to achieve optimal prevention of dental caries. The fluoride concentration of community water fluoridation typically ranges between 0.5 and 1.1 mg/l. In many countries Community water fluoridation is a core component of oral health policy [10].

An English study on the effect of water fluoridation (Roberts et al. 2022) [11] found that in 5-year-old children, the prevalence of dental caries was 6% lower in areas with 0.7 m/l of water fluoride than areas where concentrations were below 0.1 mg/l. The study also showed a 59% decrease in hospitalizations due to dental caries in individuals aged 0–19 years when fluoride levels were higher.

A study across 10 countries performed by Rugg-Gunn and Do in 2012 [12] on individuals aged between 3 and 44 years, found a 30–59% reduction of caries in the primary dentition, and a 40–49% reduction in the permanent dentition in areas with fluoridated water.

Following the recommendation of WHO, Community water fluoridation began in Israel at the municipal level in 1981, and the optimal fluoride concentration ranged between 0.7 and 1.2 PPM, depending on local temperature and water consumption. At this time, about 90% of the children suffered from tooth decay, and dental public services were limited [13]. From 2002 Community water fluoridation became mandatory, and 75% of the population received fluoridated water [14]. Even though Community water fluoridation in Israel significantly helped to reduce dental caries – 56.4% of children were free of caries in fluoridated areas and 40.6% were free of caries in non-fluoridated areas [15], the Israeli minister of Health at the time decided to stop Community water fluoridation in 2014, mainly claiming that the country should not force those who do not wish to consume water with added fluoride [16]. As evidence, data published by the Ministry of Health [17] shows that until the middle of 2014 (cessation began in August), fluoride water concentrations were optimal in most areas, while right after the cessation and until today, the concentrations have dropped significantly below the recommended level of fluoride consumption.

in June 2015, following a discussion in the Ministry of Health, it was decided that water fluoridation should be reintroduced, and an amendment to the drinking water regulations was approved in March 2016. However, budgetary regulation for the re-introduction of fluoride did not follow, and consequently drinking water is not fluoridated yet [18].

A 2022 study investigated the effect of fluoridation cessation in Israel (Tobias et al.) [13]. The study examined children aged 3–12 in geographical areas where water fluoridation ceased, and used the populations of areas that were never optimally fluoridated as a control group. The number of dental treatments in 2014–2015 did not change with age. However, between 2016 and 2019, the number of treatments increased with age (after fluoridation cessation) and almost doubled. Furthermore, the areas with optimal fluoride levels had less treatments than those with partial or no fluoridation.

### Dental treatment coverage in Israel

In 1994, the National Health Insurance Law made the State of Israel responsible for the provision of health care to all citizens. Healthcare is provided by four Health Maintenance Organizations (HMOs). However, dental treatments were not included in the initial government covered healthcare services [19]. Nowadays in Israel, dental care until age18 years old is given free of charge (preventative treatments such as dental examinations), or with minimal charge (restorative treatments such as restorations, stainless-steel crowns, and treatments under general anesthesia for children 5 years and younger), following the dental reform in 2010 which was initiated by Health Minister Yaakov Litzman [20].

From a study conducted in 2016 (Natapov et al.) [19], it seemed that the level of dental disease remained constant after the reform, yet an increase in the treatment component was observed, following the reform.

### Dental treatments given under general anesthesia

Most children can be treated adequately using behavioral techniques. However, some children require general anesthesia e.g., very young children, or those suffering from physical, mental, cognitive, or emotional immaturity or disability, or severe anxiety. General anesthesia is a controlled state of unconsciousness in which protective reflexes are lost. One of its greatest advantages is the fact that general anesthesia does not require cooperation [21].

### “Maccabi-Dent”

“Maccabi” is the second largest HMO in Israel with 2.3 million patients. “Maccabi-Dent” is the HMO’s dental clinic, and has 53 clinics and 1100 dentists [22]. In the specialist clinic “Assuta Tel Aviv”, some treatments are performed under general anesthesia or deep sedation, due to complicated dental status, or the reasons mentioned above. Patients are referred to “Assuta” clinic from different “Maccabi-Dent” clinics across the country. They come from different cities and different sociodemographic backgrounds, representing a sample of Israel’s entire population. Our study used data from treatments performed on children aged 3–5 at “Assuta Tel Aviv” between 2014 and 2019.

We hypothesized that after the cessation of water fluoridation, there may have been a greater need for dental treatments due to an increase in dental caries. We aimed to examine the effects of cessation of water fluoridation on the dental treatment needs of children aged 3–5.

## Materials and methods

The present study was approved by the Institutional Review Board (IRB) MHS 0157 − 20 The Helsinki committee of Macabi Healthcare services.

This retrospective study used data of children aged 3 to 5 years from the computerized data base of “Maccabi-Dent”, between 2014 and 2019. The database included information regarding the number of treatments performed on children aged 3 to 12, from all over the country, treated at “Maccabi-Dent” clinics. Treatments included extractions, root canal treatments, prefabricated crowns, amalgam restorations, composite restorations and more. The data was recorded as codes, each code representing a different treatment, and we only extracted codes relevant to the current study - the number of stainless-steel crowns and the number of restorations of all types (amalgam and composite resin restorations on all surfaces), performed at “Assuta Tel Aviv” branch in Ramat Hayail. The rationale for selection of these particular treatments will be outlined in the discussion.

We compared the treatments by year - from 2014 to 2019 and by age 3 to 5. Additionally, a comparison between the years where fluoridated water was present (2014–2015) and the years without fluoridated water (2016–2019) was made.

### Statistical methods

In order to compare the number of treatments before and after Community water fluoridation cessation for all age groups, a Kruskal-Wallis test (a non-parametric test which examines whether independent populations came from the same source) was performed. The Kruskal-Wallis test can only be performed when the variables between the two populations being compared are not significantly different, in this study there were relatively few parameters to compare. A two tailed test was also performed, alpha = 0.05. Python software version 3.7 was used.

## Results

### Descriptive data

The total number of treatments performed between 2014 and 2019 in children aged 3–5 was 34,606 crowns and 44,358 restorations, with means of 1922 ± 1051 crowns and 2464 ± 942 restorations in each year (dividing the sum of the mean number of treatments performed each year by the number of years). The number of treatments per year increased between 2014 and 2019 (see Table 1).

**Table 1:** Total treatments per year

Year	Total number of crowns (percentage out of total treatments)	Total number of restorations (percentage out of total treatments)	Total number of treatments (crowns and restorations)
**2014**	3121 (41.2%)	4450 (58.8%)	7571
**2015**	3588 (40.7%)	5227 (59.3%)	8815
**2016**	4585 (37.8%)	7533 (62.2%)	12,118
**2017**	5769 (41%)	8274 (59%)	14,043
**2018**	7536 (45.3%)	9085 (54.7%)	16,621
**2019**	10,007 (50.5%)	9789 (49.5%)	19,796

**Fig. 1:**
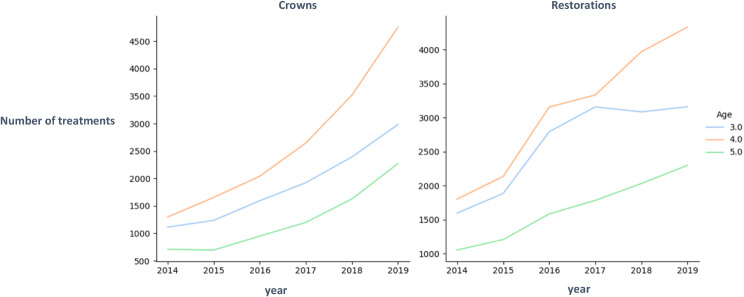
Treatments per age and year

Figure 1 shows the number of treatments performed by age and by year. An upward trend in all age groups, especially regarding the number of crowns can be seen. Most treatments doubled over the years, and the increase in 4-year-olds was the greatest. More restorations than crowns were performed in 3-year-olds, whereas this trend was reversed in 4-year-olds. 5-year-olds had the lowest number of treatments.

In 2014–2015 the patients had been drinking fluoridated water whereas those treated between 2016 and 2019 were drinking water without fluoride. With fluoride 6709 crowns and 9677 restorations were performed with means of 1118 ± 368, and 1612 ± 414 respectively. Without fluoride, 27,897 crowns and 34,681 restorations were performed, with means of 2324 ± 1057, 2890 ± 837 respectively.

**Fig. 2:**
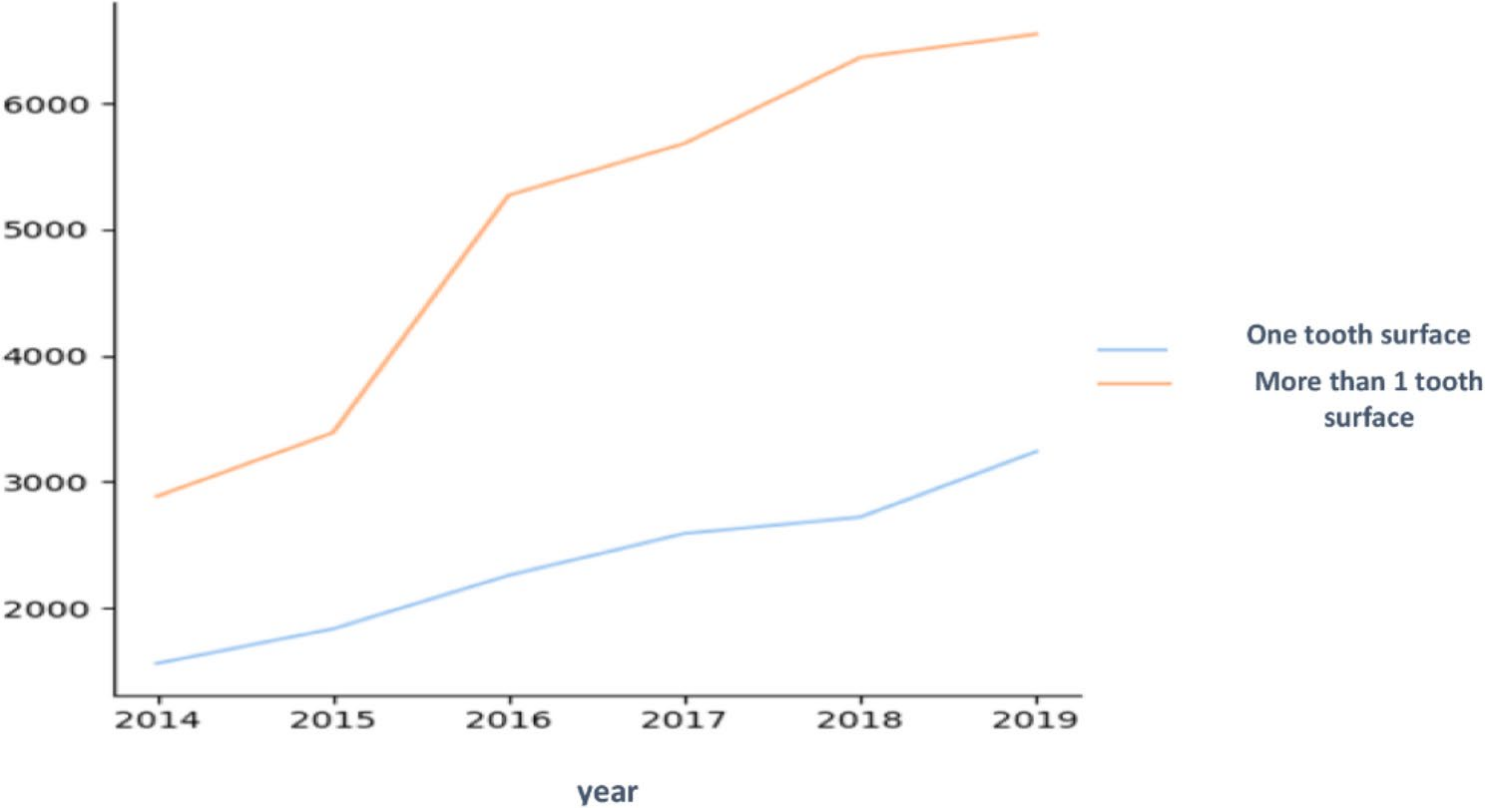
Comparison between restorations performed on one tooth surface and on more than one tooth surface - all ages

**Fig. 3:**
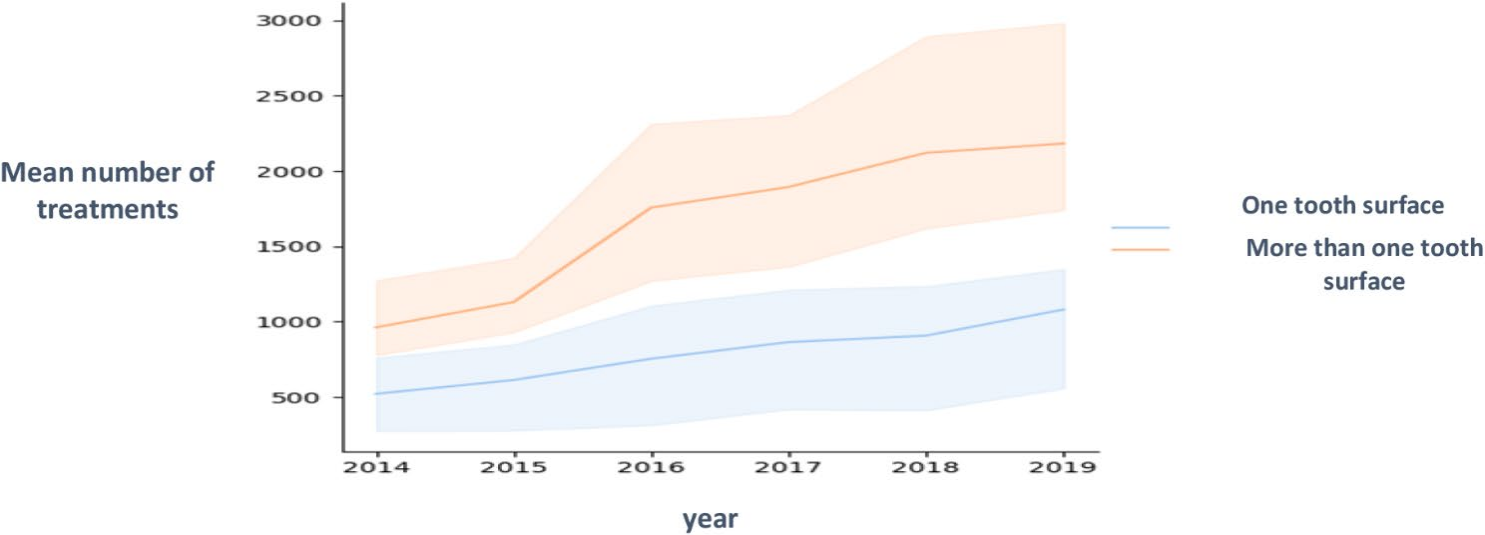
Comparison between the number of restorations performed on one tooth surface and on more than one tooth surface

**Fig. 4:**
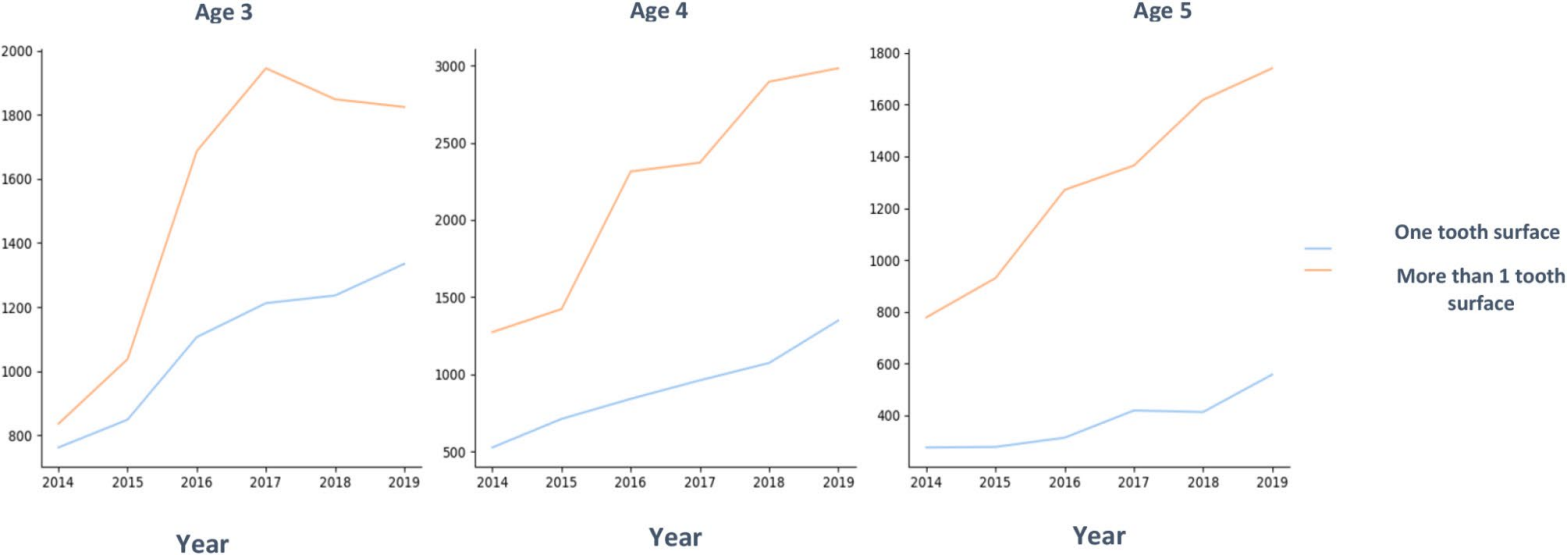
Comparison between number of restorations performed on one tooth surface and on more than one tooth surface by age

The number of restored surfaces per tooth was also compared. The number of treated surfaces is an indication of disease severity. As seen in Figs. 2, 3 and 4, there were more treatments on more than one surface, and the rate of increase of multi-surface restorations was greater than the rate of increase of single surface treatments. The greatest increase was in 4-year-olds. Figure 3 shows the statistically significant difference between the number of restorations performed on one or more tooth surfaces. 3-year-olds had the most one surface restorations and 5-year-olds had the least.

### Analytic data

**Fig. 5:**
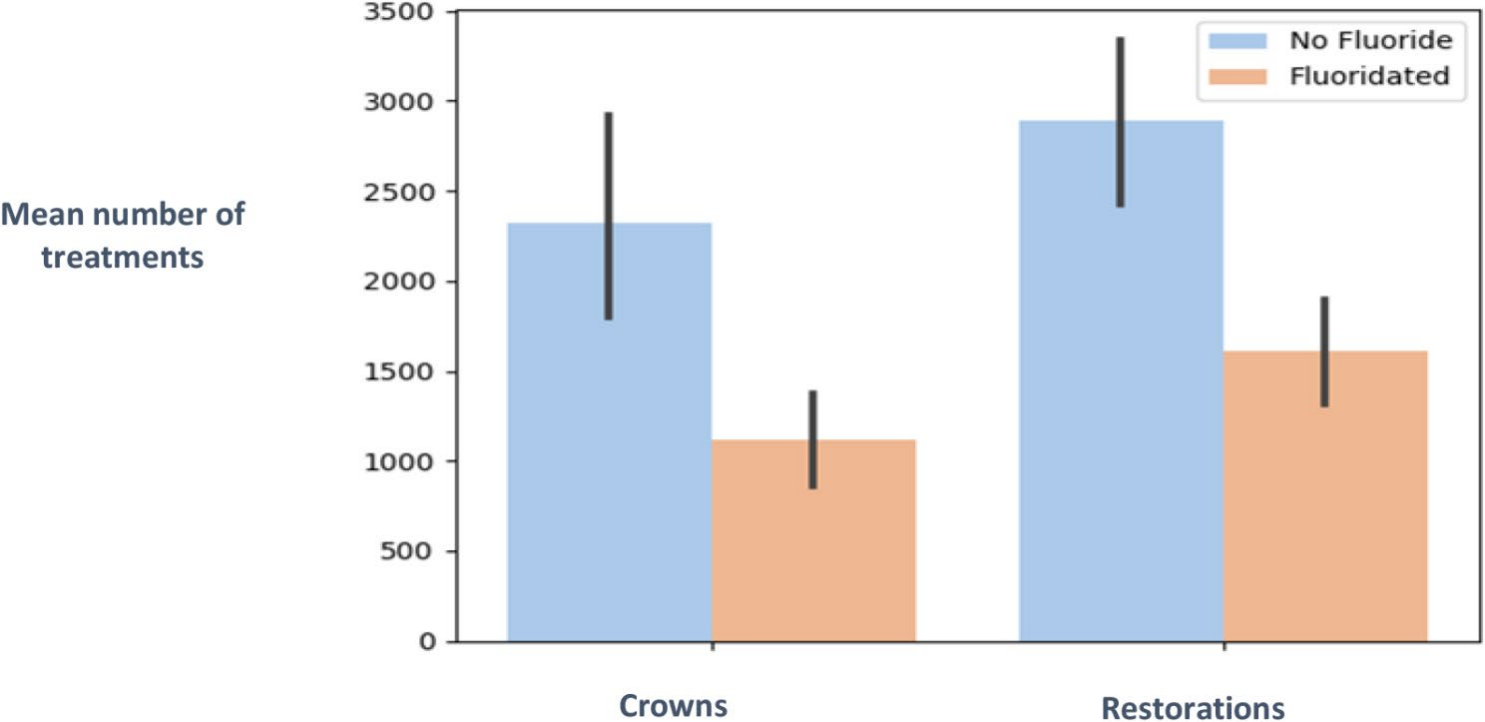
Mean number of treatments with or without fluoride

Dependent variables were the number of treatments i.e., crowns and restorations. Independent variables were age, fluoridated (2014–2015) vs. non fluoridated water (2016–2019) and year. Figure 5 shows the results of the Kruskal Wallis test. there were significantly more treatments in the years when the water was not fluoridated than when there was fluoride in the water (*p* < 0.05).

**Fig. 6:**
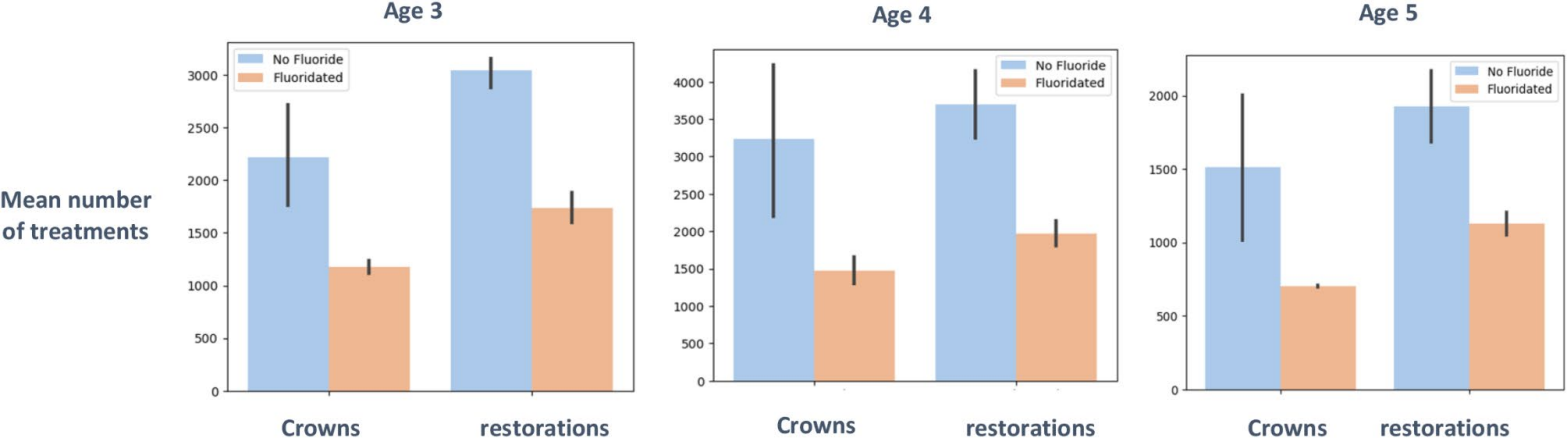
Mean number of treatments with or without fluoride by age

Figure 6 shows the mean number of treatments with and without fluoride by age. The greatest difference was in 3-year-olds before and after fluoridation cessation, especially in restorations. The Difference exists in all age groups, and the mean number of treatments almost doubled in all age groups.

## Discussion

The purpose of the current study was to examine the influence of water fluoridation cessation in Israel on the dental treatment needs of children aged 3–5. We collected computerized treatment records from 2014 to 2019 from “Assuta Tel Aviv”, a specialist dental clinics, where treatments are performed under general anesthesia or deep sedation, belonging to “Maccabi” HMO. Young children usually require deep sedation or general anesthesia [23]. Due to the fact that the specialists at “Assuta Tel Aviv” receive patients from all over the country, our study included a sample of the entire population.

We examined standard treatments for teeth with carious lesions - Restorations for smaller lesions and stainless-steel crowns for situations when much of the tooth material was lost due to extensive caries [5]. An increase in the total number of treatments or in the proportion of crowns indicates whether there has been a decline in dental health and the severity of the decline.

We noted a correlation between the number of treatments performed and fluoridation cessation, which is reflected by an almost 2-fold increase in the number of treatments over the years, both restorations and crowns.

When comparing treatments performed in different age groups, there was an upward trend at all ages, and a statistically significant difference between the mean number of treatments performed before (2014–2015) and after the cessation of fluoridation (2016–2019). It is important to note that when comparing the years when water was fluoridated and the years when water was not fluoridated, we used the mean results per year because there were 2 years of fluoridation and 4 years when water was not fluoridated.

Canadian studies by Mclaren in 2017 and 2021 [24, 25] support the findings of the current investigation. The 2021 study examined the effect of fluoridation cessation in children aged 7–8 years in Calgary, where fluoridation ceased in 2011 (i.e., the children were not exposed to fluoridated water), and used Edmonton, a fluoridated city, for comparison. The prevalence of caries was significantly higher without fluoride and worsened over time. The authors concluded that fluoridation cessation had a negative effect on the dental health of the children. The 2017 study showed a smaller increase in the prevalence of caries. It is important to note that initially, the proportion of the children in Calgary with caries was lower than in Edmonton. The amount of decay in primary teeth in Calgary began to rise by 2010, and accelerated after Community water fluoridation cessation in 2011 [26].

Our data showed the lowest number of treatments in 5-year-olds. This may be due to children being treated in general clinics, without the need for general anesthesia or deep sedation. Initially, there were more restorations than crowns, which equalized in 2019. Considering when fluoridation was stopped, those aged 5 in 2019 were not exposed to Community water fluoridation, whereas 5-year-olds born in 2015 were exposed to fluoridated water for at least 4 years. Similarly, 5-year-olds in 2018, had some exposure to fluoride, and the sharper increase in treatment numbers started here. Taken together it seems that the number of treatments equalized because the more severe caries lesions needed crowns, perhaps due to lack of exposure to fluoridated water from birth and before eruption.

Cohort studies (Singh and Spencer 2004, Singh et al. 2007) [27, 28] found that the pre-eruptive effect of fluoridated water is important for the prevention of caries and is of great significance during the first years of life, especially in pits and fissures of permanent teeth – since topical fluoride does not reach these areas. The effect is maximal when the exposure continues post eruption.

A 9 year cross-sectional study (D.H. Levy et al. 2023) [29] conducted in Israel examined the association between water fluoridation cessation and the dental reform in Israel in young adults.

The dental needs between subjects born in and before 1994 were less than those born in and after 1996. The researchers assumed that these differences were due to complete development of the permanent dentition without fluoride exposure in those born in and before 1994. In contrast, subjects born in or after 1996 were exposed to fluoride at a critical age in teeth development (0–6 years). This emphasizes the pre and post eruptive effects of fluoride. The study concluded that water fluoridation was significantly associated with a decrease in the need for caries related treatment.

There were more restorations than crowns in 3-year-olds, similarly there were more one surface treatments than multi-surface in this age group. This may reflect the natural progression of caries, that younger individuals had less severe lesions. In those aged 4–5 years there was a greater increase in restorations performed on more than one tooth surface. This may be explained by the fact that less conservative treatments are performed under general anesthesia [30], or the increased severity of the lesions is due to the teeth being less resilient. Indeed, a Cochrane review found that with water fluoridation, the severity of dental caries (measured by an 26% reduction in DMFT) decreases [31].

In summary, there is a correlation between fluoridation cessation and the number of dental treatments performed. The difference is statistically significant when comparing the average number of treatments at all ages before and after the cessation.

Exposure to fluoride is important both before and after tooth eruption, which emphasizes the need for restoring water fluoridation in Israel, to help prevent the occurrence of dental caries and reduce its severity. We recommend that fluoridation should occur in all communities and be nationwide, adjusting the concentrations to the existing natural amounts of fluoride in the water (which are different in each area of the country). As mentioned in the introduction, although water fluoridation was supposed to be re-introduced, it did not happen yet due to budgetary issues. However, according to the Ministry of Health, water fluoridation is a cost-effective method, and is estimated at five Shekels per resident a year (as of 2019) – a very low cost. Also, it saves dental care costs by reducing the need for it [32]. Because of the benefits of water fluoridation for dental health as demonstrated in this article and in many other articles, and due to the cost-effectiveness, we hope that this paper will help the decision makers take this important matter into account and to re-introduce water fluoridation as planned, investing in prevention and not only in dental care.

### Limitations

1. At “Assuta Tel Aviv”, most treatments are performed following a referral based on a dental examination in another branch. Therefore, we could not calculate the number of treatments in relation to the number of examinations. However, considering that the number of patients and dentists has not changed over the years, the increase in the number of treatments does not depend on the number of patients treated in this case i.e., more treatments per patient.
2. During 2014–2019, a few more “Maccabi” clinics were opened and may have referred more patients to “Assuta Tel Aviv” clinic. Nevertheless, the increase in their number was not significant, so we believe that it did not affect the results.
3. As with all retrospective cross-sectional studies, only correlations can be drawn. There was no direct causal relationship that the removal of Community water fluoridation was the fundamental reason that there were higher rates of caries and therefore more treatments. However, because many studies noted the same correlation, we can conclude that there are benefits to water fluoridation, which have been known for many years.
4. There is a lack of data regarding the “real world” exposure to fluoride water in the studied population before and after the cessation, and the extent of households in Israel that used filters. However, to the best of our knowledge, the basic natural fluoride water exposure in Israel (after the cessation) is below the recommended level of fluoride consumption.
5. Other treatments such as extractions and root canal treatments, also indicate the severity of the dental disease. Therefore, we recommend that future research should include these treatments in addition to restorations and crowns in relation to fluoridation cessation to receive more information.

## Conclusions

The results of this study emphasize the need to restore community water fluoridation and highlight the clear evidence to the decision makers regarding the necessity of water fluoridation.

## Data Availability

The data that support the findings of this study are available from “Maccabi-Dent”, but restrictions apply to the availability of these data, which were used under license for the current study, and so are not publicly available. Data are however available from the authors upon reasonable request and with permission of “Maccabi-Dent”.

## Declarations

### Healthcare services

The study was retrospective, based on medical records without any identifying information, therefore no written informed consent was needed to be obtained.
